# Identification of predictive factors for better outcomes in LINAC-based radiation treatment for cerebral cavernous malformation

**DOI:** 10.1016/j.heliyon.2024.e31184

**Published:** 2024-05-13

**Authors:** Sang Mook Kang, Byeong Jin Ha, Jin Hwan Cheong, Je Il Ryu, Yu Deok Won, Myung-Hoon Han

**Affiliations:** Department of Neurosurgery, Hanyang University Guri Hospital, 153 Gyeongchun-ro, Guri 471-701, Gyonggi-do, South Korea

**Keywords:** Cavernous malformation, Nidus volume reduction, Perilesional brain edema, Linear accelerator (LINAC)-Based radiation

## Abstract

The effectiveness of radiation therapy in the treatment of cerebral cavernous malformations (CCM) remains debatable. However, numerous studies have shown a reduction in hemorrhage risk following radiotherapy for CCM. Therefore, herein, we share our experiences utilizing linear accelerator (LINAC)-based radiation for treating CCMs, with the aim of identifying the key factors influencing the therapeutic outcomes. We conducted a retrospective review of all patients with non-brainstem CCMs who underwent radiation treatment, as recorded in the NOVALIS registry at our institution. T2-weighted MR images were used for volumetric assessments using the iPlan radiotherapy planning software. To determine the independent predictors of nidus volume reduction and perilesional brain edema (PBE), we performed multivariate Cox regression analysis to calculate hazard ratios. Twenty patients with 31 non-brainstem CCMs were enrolled in this study. Analysis revealed age as an independent predictive factor for both nidus volume reduction and PBE after radiation treatment for CCM. Furthermore, a single fraction dose of 17 Gy or more was identified as an independent predictor of nidus volume decrease, while a single fraction dose of 18 Gy or more was found to be an independent risk factor for PBE in patients with CCM following LINAC-based radiation therapy. LINAC-based radiation therapy for non-brainstem CCMs with a single fraction radiation dose between 16.5 and 17.5 Gy, or a biologically equivalent dose of approximately 120 Gy, may be the most effective at reducing nidus volume and limiting side effects, particularly in patients under the age of 55 years. We further observed that the risk of PBE increased as the maximum radiation dose delivered to a 1 cc volume of the surrounding normal brain exceeded approximately 17.3 Gy. Therefore, we believe that calculating the D_1cc_ of the normal brain may help to predict the occurrence of PBE when radiotherapy is administered to non-brainstem CCMs.

## 1 Introduction

Cerebral cavernous malformations (CCM) are low-flow vascular anomalies that commonly coexist with developmental venous anomalies and capillary telangiectasias [1]. The prevalence of CCM in the general population is relatively low, with the incidence rate reported to range between 0.3 % and 0.6 % in extensive autopsy series and prospective cohort studies [2]. The detection of CCM has increased in recent years, which is largely attributed to the more frequent use of magnetic resonance imaging (MRI) in medical settings. Although approximately 40 % of CCM cases are asymptomatic, some affected individuals experience neurological symptoms, including seizures (occurring in 23–50 % of patients), headaches (6–52 %), focal neurological deficits (20–45 %), and cerebral hemorrhages (9–56 %) [2].

CCMs have a bleeding tendency, and symptomatic hemorrhage is the most serious complication that can result in significant disability or even fatality [3]. Consequently, the main goals of CCM management involve preventing recurrent bleeding and the complications associated with hemorrhage. Surgical excision is considered the optimal treatment for CCMs. However, over the past 20 years, radiation treatment has played an increasingly important role in the treatment of deep-seated or eloquently situated CCMs [4]. Nevertheless, the efficacy of radiation treatment for CCM remains controversial, particularly in regards to the primary goal of reducing the hemorrhage rate. However, many studies have reported that cell proliferation, hyalinization, and thinning of vessel walls can occur after radiotherapy for CCM, leading to lumen closure, and thus decreasing the bleeding risk [[5], [6], [7], [8], [9], [10]].

Owing to the potential for temporary or lasting adverse effects from radiation, careful patient selection, identification of risk factors, and precise dose determination are crucial to maximize the therapeutic benefits and minimize adverse outcomes in radiation treatment for CCM [3]. Working under the knowledge that CCM lesions that decrease in size may have a low hemorrhage rate [11], we investigated the important factors that lead to CCM nidus volume reduction and a lower risk of perilesional brain edema (PBE). Herein, we present our experience with 20 patients with 31 non-brainstem CCMs treated with linear accelerator (LINAC)-based radiation to identify the crucial factors influencing treatment outcomes.

## 2 Material and methods

### 2.1 Study patients

As mentioned previously, the NOVALIS registry was established to facilitate prospective investigation of patients undergoing LINAC-based radiation therapy at our hospital [12,13]. For this study, we retrospectively extracted the data of all consecutive patients with non-brainstem CCMs who were treated with radiotherapy between July 7, 2014, and April 30, 2022 from the NOVALIS registry database at our hospital. As the efficacy of radiation treatment for CCM remains controversial, in our centers only patients with CCM accompanied by neurological symptoms, such as persistent headache or dizziness, repeated seizures even after antiepileptic drug treatment, or symptomatic cerebral bleeding, undergo radiotherapy. We limited our study to patients with CCMs who underwent at least one follow-up MRI following LINAC-based radiation therapy to evaluate changes in nidus volume and the development of PBE. All imaging results were validated by a skilled neuroradiologist. Additionally, we examined the duration of follow-up imaging for all participants after treatment.

This study was approved by the Institutional Review Board of Hanyang University Guri Hospital, South Korea, and conformed to the tenets of the Declaration of Helsinki. The need for informed consent was waived due to the retrospective nature of the study. All individual records were anonymized prior to analysis.

### 2.2 Radiation technique

The radiation technique employed at our facility has been previously documented [12,13]. In brief, patients with non-brainstem CCMs at our institution received treatment using the NOVALIS Tx system (Varian Medical Systems, CA, USA; Brainlab, Feldkirchen, Germany). Noninvasive thermoplastic masks are used to facilitate simulated computed tomography (CT) for radiotherapy planning. To increase the accuracy of radiation delivery, we utilized the Novalis ExacTrac image system and a robotic couch to adjust patient positioning based on real-time image data. Treatment involved 6 MV LINAC-based radiation administered within one week of the CT simulation.

**Fig. 1:**
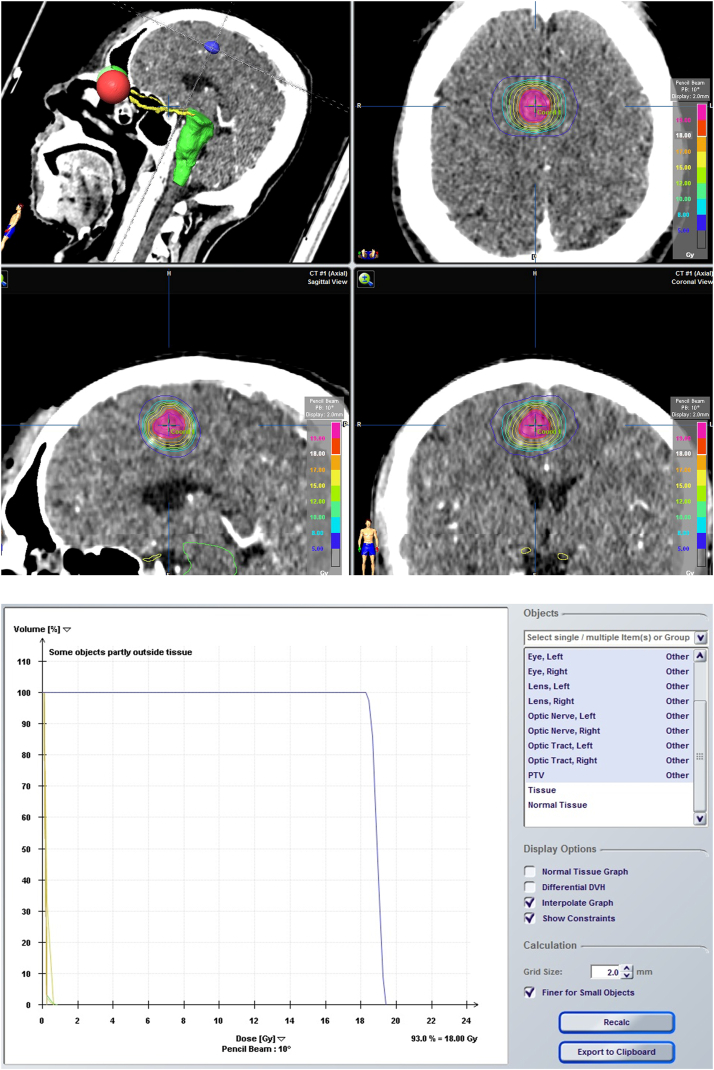
Isodose line covering and cumulative dose-volume histogram for the radiation treatment for CCM performed with the NOVALIS Tx system (Varian Medical Systems, CA, USA; Brainlab, Feldkirchen, Germany). CCM, cerebral cavernous malformation.

For radiation treatment planning, the iPlan (Brainlab, Feldkirchen, Germany) and Eclipse (Varian, CA, USA) 3D treatment/planning systems of the NOVALIS Tx were employed on the MRI/CT fusion images for all patients with CCM. The 3D treatment planning system automatically determined the target volume of the CCM nidus. Our objective was to achieve precise conformity of the treatment isodose with the 3D reconstructed geometry of the CCM nidus to cover the margin of the lesion (Fig. 1). Stereotactic radiosurgery (SRS) was administered as a single-session treatment, or in hypofractionated sessions of two–five fractions. To facilitate dose comparison across patients with different fractionation schedules, we calculated the biologically equivalent dose (BED) for the tumor using the formula:

BED = nd × (1 + d/3),

where n represents the number of fractions, d denotes the dose per fraction, and α/β equals 3.

### 2.3 Image analysis and outcome assessment

All MRI scans, with slice thicknesses ranging from 1.5 to 3.0 mm, were performed using the Ingenia 3.0 T CX (Philips Ingenia, Philips Medical Systems, Böblingen, Germany) and Achieva 3.0 T TX (Philips Achieva, Philips Medical Systems, Böblingen, Germany) systems at our facility. According to our hospital protocol, following LINAC-based radiation treatment, unless neurological symptoms develop, patients are usually followed up with MRI approximately once every six months for the first two years after radiation. We performed volumetric analysis of T2-weighted MR images using the iPlan radiotherapy planning software, and further measured the whole brain volume to calculate the D_1cc_ (the maximum dose delivered to a volume of 1 cc of the surrounding normal brain parenchyma; the following two parameters are similar), D_10cc_, and D_15cc_ values in all patients with CCM.

CCM lesions were classified based on MRI characteristics using the Zabramski classification system [14]. Changes in CCM nidus volume were evaluated as follows: an increase of 20 % or more was categorized as an increase, a decrease of 20 % or more was categorized as a decrease, and changes in volume of less than 20 % from the baseline were considered stable [2]. PBE was defined as new-onset high signal intensity on T2-weighted MRI. Antiepileptic drugs were immediately administered to all patients with CCM who experienced seizures. Symptomatic cerebral hemorrhage was defined as an obvious cerebral hemorrhage based on radiographic images and clinical symptoms such as headache, disturbance of consciousness, seizures, and neurological deficits.

### 2.4 Statistical analysis

Continuous variables are reported as the means ± standard deviation (SD) or median with interquartile range (IQR), while categorical variables are presented as counts and percentages. Student's t-test was used to determine statistical differences in continuous variables across two distinct groups.

Receiver operating characteristic (ROC) curve analysis was performed to identify the optimal cutoff values for predictive factors of nidus volume reduction and PBE following radiation treatment in CCMs. The ideal cut-off value was established as the point closest to the upper-left corner of the curve. The proximity of each point on the ROC curve to this corner was measured using the formula:

$$\sqrt{{\left(1\;–\;\text{sensitivity}\right)}^{2}+{\left(1\;–\;\text{specificity}\right)}^{2}}\text{.}$$

The cumulative rates of nidus volume reduction or PBE were computed using Kaplan-Meier analysis, and classified based on various predictive factors. To identify independent predictive factors for nidus volume reduction and PBE, hazard ratios (HRs) with 95 % confidence intervals (CIs) were calculated using univariate and multivariate Cox regression analyses.

A scatterplot with a linear regression line was used to demonstrate the relationship between patient age and either the BED or the single-fraction radiation dose, depending on the occurrence of PBE after radiation therapy for CCM.

Statistical significance was set at P < 0.05. All statistical procedures were conducted using R software version 4.2.2 and SPSS for Windows (version 24.0 (IBM, Chicago, IL, USA).

## 3 Results

### 3.1 Characteristics of the study patients

This study included 20 patients with 31 CCMs, all of whom received LINAC-based radiation therapy at our hospital over approximately eight years. The average age of the patients at the time of radiation treatment was 49.8 years, and 45.0 % were women (Table 1). The median time from radiation treatment to final imaging follow-up was 28.5 months. Seizures were the most frequent initial symptoms, reported in 50.0 % of cases, and 20.0 % of the patients presented with multiple CCMs. The average volume of the CCMs targeted during radiation and the BED with an α/β ratio of 3 were 2.5 cc and 109.2 Gy, respectively. At the last follow-up, the mean volume of the CCMs decreased to 1.8 cc. Additional details of patient characteristics are presented in Table 1.

**Table 1:** Characteristics of patients with CCM who underwent LINAC-based radiation treatment.

Characteristics	Total
Number of patients	20
Total number of CCMs	31
Sex, female, n (%)	9 (45.0)
Age at radiation treatment for CCM, mean ± SD, y	49.8 ± 15.6
Time interval between radiation treatment and the last follow-up image, median (IQR), months	28.5 (16.0–37.8)
Initial presenting symptoms, n (%)	
Intractable headache and/or dizziness	6 (30.0)
Seizure	10 (50.0)
Hemiparesis due to cerebral hemorrhage	1 (5.0)
Other neurological deficits	3 (15.0)
Number of CCM lesions, n (%)	
Single	16 (80.0)
Multiple	4 (20.0)
Locations of CCM, n (%)	
Frontal	8 (25.8)
Temporal	8 (25.8)
Parietal	11 (35.5)
Occipital	1 (3.2)
Cerebellum	3 (9.7)
Zabramski classification, n (%)	
I	3 (9.7)
II	21 (67.7)
III	3 (9.7)
IV	0
V	4 (12.9)
CCM target volume at radiation treatment, mean ± SD, cc	2.5 ± 3.5
CCM volume at the last follow-up MR image after radiation, mean ± SD, cc	1.8 ± 2.5
Marginal radiation dose, mean ± SD, Gy	20.1 ± 5.6
Fractionation, n (%)	
SRS (single fraction)	23 (74.2)
hypofractionated -SRS (2–5 fractions)	8 (25.8)
BED (α/β = 3), mean ± SD, Gy	109.2 ± 19.3

CCM, cerebral cavernous malformation; LINAC, linear accelerator; SD, standard deviation; IQR, interquartile range; SRS, stereotactic radiosurgery; BED, biologically equivalent dose.

**Fig. 2:**
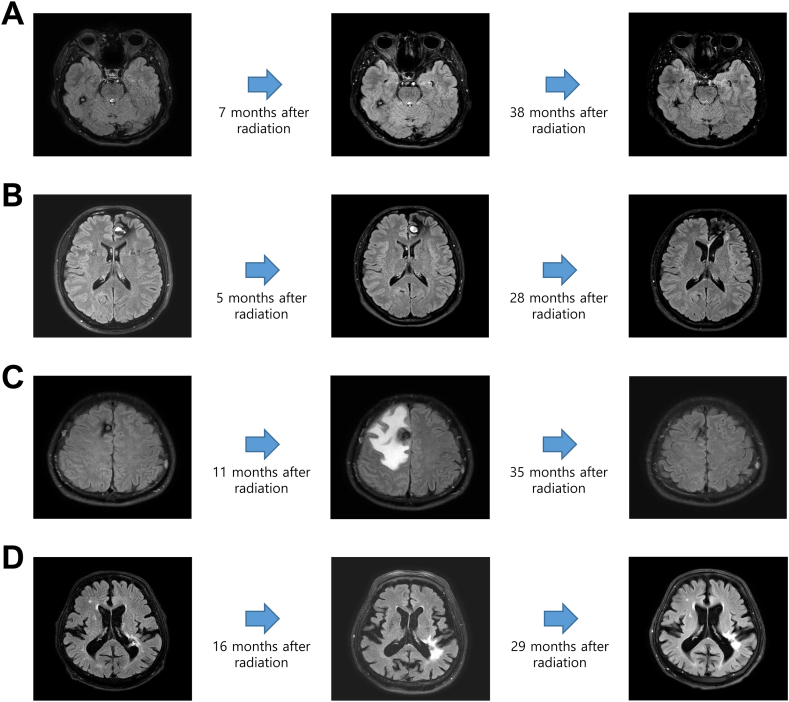
(A) A 31-year-old male patient suddenly developed blurred vision. MRI revealed a CCM in the right temporal area and SRS treatment with 18 Gy was administered. Nidus volume reduction was observed 38 months after SRS treatment; (B) A 39-year-old male patient presenting with repeated seizure attacks. MRI revealed a cystic CCM in the left frontal lobe and SRS treatment with 18 Gy was administered. Nidus volume reduction was observed 28 months after SRS treatment; (C) A 45-year-old male patient presenting with persistent headache and dizziness. MRI revealed a CCM in the right high frontal lobe and SRS treatment with 18 Gy was administered. Eleven months after radiation treatment, the patient presented with left hemiparesis and was admitted. MRI revealed an intranidal hemorrhage with severe PBE. The patient was treated with steroids for 4 months to control the brain edema. Thirty-five months after SRS treatment, the patient recovered completely and the last follow-up MRI showed CCM nidus volume decrease and no PBE. (D) An 81-year-old male patient presenting hemiparesis due to cerebral hemorrhage. MRI revealed a CCM located in the left posterior paraventricular area and SRS treatment with 17 Gy was administered. Sixteen months after SRS treatment, follow-up MRI showed nidus volume decrease with asymptomatic PBE. The patient was treated with steroid for 3 months. Twenty-nine months after radiation treatment, the patient's symptoms improved and the last follow-up MRI showed CCM nidus volume decrease and persistent but slightly decreased asymptomatic PBE. CCM, cerebral cavernous malformation; MRI, magnetic resonance imaging; PBE, perilesional brain edema; SRS, stereotactic radiosurgery.

Fig. 2A–D presents serial follow-up MR images (FLAIR) of the progression of several patients who underwent LINAC-based radiation treatment for CCM. More detailed follow-up MR scans, including T1-and T2-weighted images, are shown in Supplementary Figs. 1–4.

### 3.2 Outcomes after LINAC-based radiation treatment for CCM

Nidus volume reduction was achieved in 64.5 % of patients, while PBE occurred in 25.5 % of CCM patients after radiotherapy during the follow-up period (Table 2). Symptomatic cerebral hemorrhage was not observed after radiotherapy for CCM; however, asymptomatic intranidal hemorrhage was present in 22.6 % of the 31 CCMs cases in our study (mostly accompanied by PBE). Overall symptom improvement was observed in 80 % of patients (Table 2).

**Table 2:** Outcomes after LINAC-based radiation treatment for CCM.

Outcomes after LINAC-based radiation treatment for CCM	
Nidus volume reduction (volume reduction by more than 20 %), n/total n (%)	20/31 (64.5)
Perilesional edema, n/total n (%)	8/31 (25.8)
Symptomatic cerebral hemorrhage, n/total n (%)	0/31 (0)
Insignificant intranidal hemorrhage, n/total n (%)	7/31 (22.6)
Developed neurological deficits after radiation, n/total n (%)	6/20 (30.0)
Transient, n/total n (%)	5/20 (25.0)
Permanent, n/total n (%)	1/20 (5.0)
Patients with newly developed CCMs after radiation therapy, n/total n (%)	2/20 (10.0)
Overall symptom improvement, n/total n (%)	16/20 (80.0)
Improvement of intractable headache and/or dizziness, n/total n (%)	4/6 (66.7)
Improvement of hemiparesis and no further cerebral hemorrhage, n/total n (%)	1/1 (100)
Control of epileptic symptoms, n/total n (%)	9/10 (90.0)
Improvement of other neurological deficits, n/total n (%)	2/3 (66.7)

LINAC, linear accelerator; CCM, cerebral cavernous malformation.

Optimal radiation dose for predicting nidus volume reduction and PBE after radiation for CCM.

**Fig. 3:**
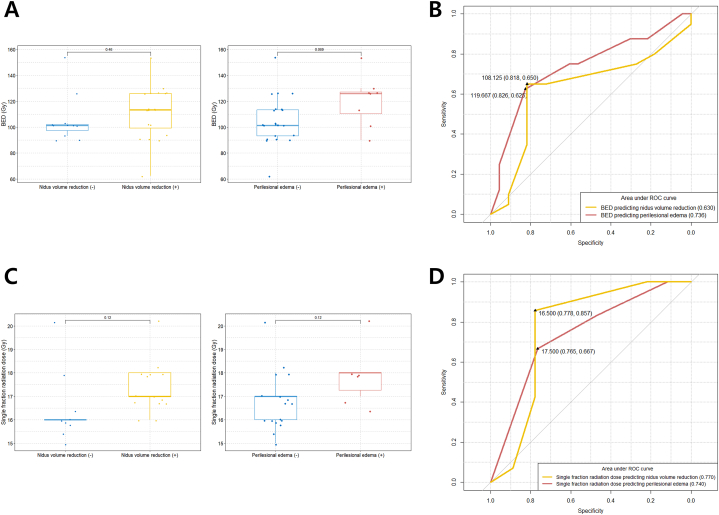
(A) Comparison of BED between nidus volume reduction (+) and nidus volume reduction (−) groups and PBE (+) and PBE (−) groups; (B) Determination of the optimal cut-off BED values for predicting nidus volume reduction and PBE; (C) Comparison of single fraction radiation dose between nidus volume reduction (+) and nidus volume reduction (−) groups and PBE (+) and PBE (−) groups; (D) Determination of the optimal cut-off single fraction radiation dose values for predicting nidus volume reduction and PBE. BED, biologically equivalent dose; PBE, perilesional brain edema.

Although not statistically significant due to the small sample size, we observed that the higher the BED, the higher the likelihood of both nidus volume reduction and the development of PBE after LINAC-based radiation therapy for CCM (Fig. 3A). The optimal BED cutoff values for predicting nidus volume reduction and PBE after radiotherapy for CCM were 108.125 Gy (Area Under the Curve [AUC], 0.630; sensitivity, 65.0 %; specificity, 81.8 %) and 119.667 Gy (AUC, 0.736; sensitivity, 62.5 %; specificity, 82.6 %), respectively (Fig. 3B).

Similarly, although not statistically significant, the SRS group showed higher rates of nidus volume reduction and PBE as the radiation dose increased (Fig. 3C). We further observed that the optimal single fraction radiation dose values for predicting nidus volume reduction and PBE after radiation treatment for CCM were 16.5 Gy (AUC = 0.770; sensitivity = 85.7 %; specificity = 77.8 %) and 17.5 Gy (AUC = 0.740; sensitivity = 66.7 %; specificity = 76.5 %), respectively (Fig. 3D).

### 3.3 Cumulative rates of nidus volume reduction and PBE after radiation according to several predictive factors

**Fig. 4:**
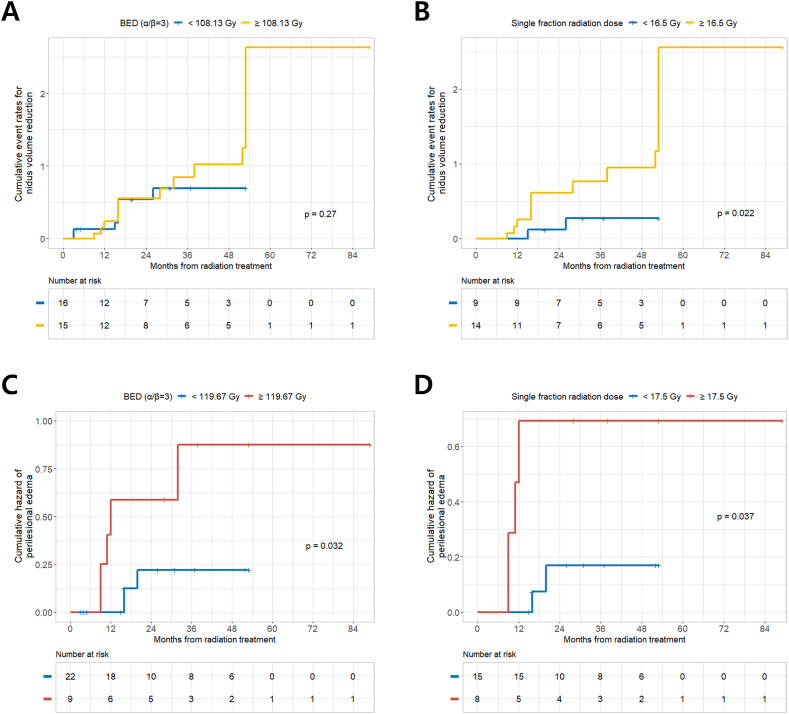
(A) Cumulative event rates for nidus volume reduction after LINAC-based radiation treatment for CCM according to the cut off value of BED (108.13 Gy); (B) Cumulative event rates for nidus volume reduction according to the cut off value of single radiation dose (16.5 Gy); (C) Cumulative hazard of PBE according to the cut off value of BED (119.67 Gy); (D) Cumulative hazard of PBE according to the cut off value of single radiation dose (17.5 Gy). BED, biologically equivalent dose; CCM, cerebral cavernous malformation; LINAC, linear accelerator; PBE, perilesional brain edema.

We classified CCM lesions based on the established cutoff values of the BED (108.13 Gy) and SRS radiation doses (16.5 Gy) to predict nidus volume reduction after LINAC-based radiation therapy for CCM. While the higher BED group showed no statistically significant CCM nidus volume reduction, SRS radiation doses >16.5 Gy were found to be significantly associated with CCM nidus volume reduction (p = 0.022) (Fig. 4A and B).

Additionally, to identify the relationship between the occurrence of PBE and radiation dose after radiation therapy for CCM, we classified CCM lesions according to the cutoff values of the BED (119.67 Gy) and SRS radiation doses (17.5 Gy) for the prediction of PBE. We observed that CCMs that received higher BED (≥119.67 Gy) and SRS radiation dose (≥17.5 Gy) showed a significantly higher incidence of PBE during the clinical course of CCM following LINAC-based radiation treatment (Fig. 4C and D).

Independent predictive factors of nidus volume reduction and PBE after radiation in patients with CCM.

Multivariate Cox regression analysis of all CCM lesions (n = 31) revealed that younger age was an independent factor predicting a reduction in nidus volume following LINAC-based radiation therapy, with an HR of 0.96 and a significance level (p) of 0.037 (Table 3). When multivariate Cox regression analysis was conducted on PBE occurrence in CCM, the risk of PBE was significantly higher in the older age and high BED (≥119.67 Gy) groups. The results of univariate Cox regression analysis of nidus volume reduction and PBE in the CCM are shown in Supplementary Table 1.

**Table 3:** Multivariate Cox regression analyses of nidus volume reduction and perilesional brain edema after LINAC-based radiation treatment for CCM based on predictive factors in all patients and patients received SRS.

All CCMs (n = 31)				
	Nidus volume reduction		Perilesional brain edema	
Variable	HR (95 % CI)	p	HR (95 % CI)	p
Sex				
Male	Reference		Reference	
Female	0.57 (0.17–1.85)	0.348	0.54 (0.07–4.33)	0.564
Age (per 1-year increase)	**0.96 (0.92**–**1.00)**	**0.037**	**1.13 (1.01**–**1.27)**	**0.036**
Location				
Supratentorial	Reference		Reference	
Infratentorial	2.30 (0.36–14.67)	0.380	0.07 (0.00–2.08)	0.126
BED				
<108.13 Gy	Reference		N/A	
≥108.13 Gy	3.07 (0.82–11.50)	0.095		
<119.67 Gy	N/A		Reference	
≥119.67 Gy			**85.42 (2.55**–**2857.97)**	**0.013**
Zabramski classification	1.28 (0.62–2.62)	0.507	1.07 (0.32–3.58)	0.912
CCM volume (per 1 cc increase)	0.97 (0.78–1.21)	0.769	1.46 (0.98–2.16)	0.063
Fractionation				
SRS	Reference		Reference	
hf-SRS	4.52 (0.97–21.10)	0.055	0.31 (0.02–5.39)	0.420
**CCMs received single fraction radiotherapy (n=23)**				
	Nidus volume reduction		Perilesional brain edema	
Variable	HR (95 % CI)	p	HR (95 % CI)	p
Sex				
Male	Reference		Reference	
Female	0.35 (0.07–1.80)	0.207	0.36 (0.03–3.79)	0.395
Age (per 1-year increase)	0.96 (0.91–1.00)	0.060	1.11 (1.00–1.23)	0.054
Location				
Supratentorial	Reference		Reference	
Infratentorial	3.47 (0.38–31.59)	0.270	0.23 (0.01–5.35)	0.359
Radiation dose				
<16.5 Gy	Reference		N/A	
≥16.5 Gy	**34.09 (1.18**–**985.73)**	**0.040**		
<17.5 Gy	N/A		Reference	
≥17.5 Gy			**80.19 (2.62**–**2452.37)**	**0.012**
CCM volume (per 1 cc increase)	2.18 (0.78–6.13)	0.140	2.83 (0.79–10.18)	0.111

LINAC, linear accelerator; CCM, cerebral cavernous malformation; SRS, stereotactic radiosurgery; HR, hazard ratio; CI, confidence interval; BED, biologically equivalent dose; N/A, not available.

**Fig. 5:**
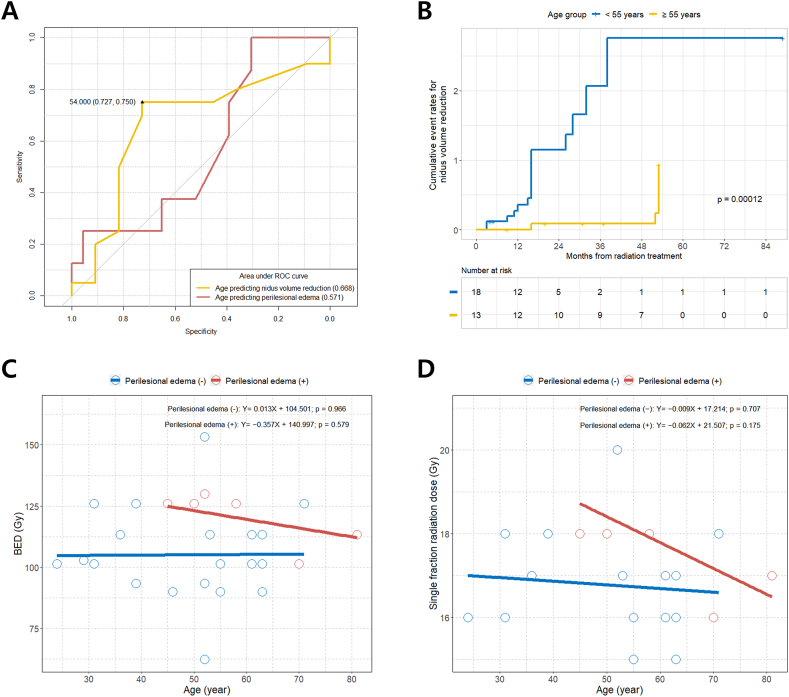
(A) Determination of the optimal cut-off age for predicting nidus volume reduction and PBE after LINAC-based radiation treatment for CCM; (B) Cumulative event rates for nidus volume reduction according to the cut off value of age (55 years); (C) Scatter plot with linear regression lines showing the associations between age and BED according to PBE presence/absence; (D) Scatter plot with linear regression lines showing the associations between age and single fraction radiation dose according to PBE presence/absence. BED, biologically equivalent dose; CCM, cerebral cavernous malformation; LINAC, linear accelerator; PBE, perilesional brain edema.

We found that the cut-off age for predicting nidus volume reduction after radiation treatment for CCM was 54 years (AUC, 0.668; sensitivity, 75.0 %; specificity, 72.7 %) (Fig. 5A). When we classified CCM lesions based on a cut-off age of 54 years, the younger age group (<55 years) showed a significant nidus volume reduction rate than the older age group after LINAC-based radiation therapy for CCM (Fig. 5B). Although not statistically significant due to the small sample size, we observed that as age increased, the PBE of CCM could occur even with a relatively low BED and single-fraction radiation dose (Fig. 5C and D).

In contrast, in patients with CCM lesions who received SRS treatment (n = 23), only the radiation dose was found to be an independent predictive factor, showing a statistically significant association with both nidus volume reduction (≥16.5 Gy) and PBE (≥17.5 Gy) in multivariate Cox regression analysis (HR, 34.09; p = 0.040; HR, 80.19; p = 0.012, respectively) (Table 3).

The dose received by the normal brain parenchyma is associated with PBE after single fraction radiation treatment for CCM.

**Fig. 6:**
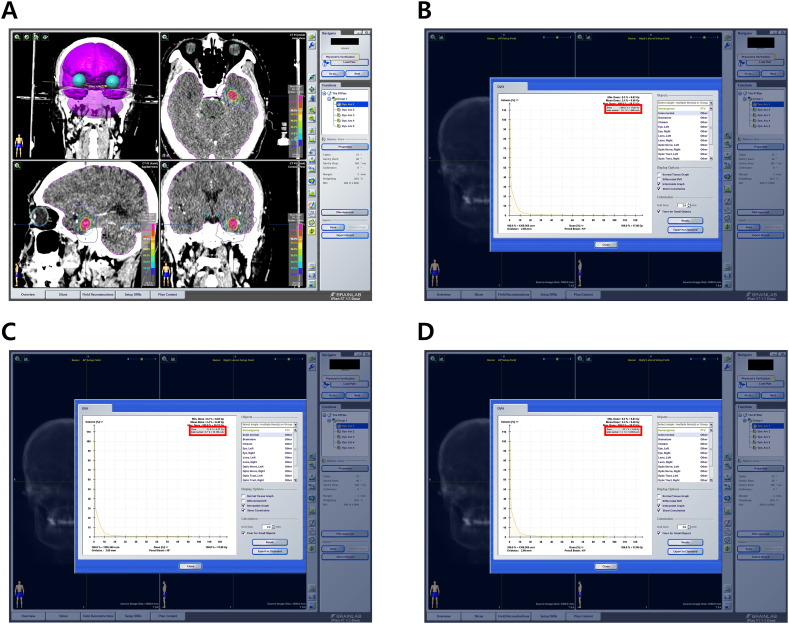
(A) Isodose line covering a nonbrainstem CCM and whole brain volume measurement using the iPlan BrainLab planning system; (B) Cumulative dose-volume histogram for the calculation of D_1cc_ in a normal brain; (C) Cumulative dose-volume histogram for the calculation of D_10cc_ in a normal brain; (D) Cumulative dose-volume histogram for the calculation of D_15cc_ in a normal brain. CCM: cerebral cavernous malformation; D_1cc_: maximum dose delivered to a volume of 1 cc of surrounding normal brain parenchyma; D_10cc_: maximum dose delivered to a volume of 10 cc of surrounding normal brain parenchyma; D_15cc_: maximum dose delivered to a volume of 15 cc of surrounding normal brain parenchyma.

**Fig. 7:**
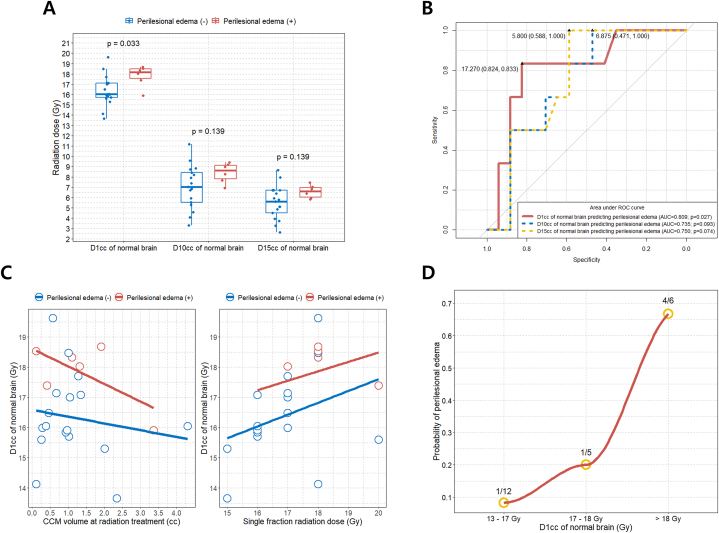
(A) Boxplots of D_1cc_, D_10cc_, and D_15cc_ distributions according to PBE presence/absence after LINAC-based radiation treatment for nonbrainstem CCM; (B) Determination of optimal cut-off values for D_1cc_, D_10cc_, and D_15cc_ of normal brain parenchyma for predicting PBE occurrence; (C) Scatter plot with linear regression lines showing the association between CCM volume during radiation treatment and D_1cc_ of normal brain parenchyma (left) and between single fraction radiation dose and D_1cc_ of normal brain parenchyma (right) according to PBE presence/absence; (D) LOWESS regression curve based on intervals of D_1cc_ of normal brain and the probability of PBE. For each, D_1cc_ < 17 Gy interval, D_1cc_ between 17 Gy and 18 Gy interval, and D_1cc_ > 18 Gy interval, the PBE incidence rate is displayed. CCM, cerebral cavernous malformation; D_1cc_, maximum dose delivered to a volume of 1 cc of surrounding normal brain parenchyma; D_10cc_, maximum dose delivered to a volume of 10 cc of surrounding normal brain parenchyma; D_15cc_, maximum dose delivered to a volume of 15 cc of surrounding normal brain parenchyma; LINAC, linear accelerator; LOWESS, locally weighted scatterplot smoothing; PBE, perilesional brain edema.

We further analyzed whether the dose received by the normal brain parenchyma following radiotherapy for non-brainstem CCM had a significant effect on the occurrence of PBE. Fig. 6A–D shows the D_1cc_, D1_0cc_, and D_15cc_ values of the surrounding normal brain parenchyma calculated using the planning system for the study patient. Overall, we found a significant difference in the D_1cc_ of the normal brain between the PBE (+) and PBE (−) groups after single-fraction radiation treatment for CCM (p = 0.033) (Fig. 7A). The optimal cut-off value of the D_1cc_ of the normal brain to predict PBE after CCM single fraction radiotherapy was 17.27 Gy (AUC = 0.809; sensitivity = 83.3 %; specificity = 82.4 %; p = 0.027) (Fig. 7B). We further found that a higher D_1cc_ in the normal brain was associated with a higher incidence of PBE when radiation was delivered at a similar CCM volume (Fig. 7C). Similarly, when the same single-fraction radiation dose was delivered to the CCM, a higher D_1cc_ value in the normal brain was associated with a higher risk of PBE (Fig. 7C). We further present the results of the relationship between D_10cc_ and D_15cc_ in the normal brain and the CCM volume and single-fraction radiation dose in Supplementary Fig. 5. When patients were categorized between 17 and 18 Gy, based on the D_1cc_ of the normal brain, the incidence of PBE increased more than 2-fold when the D_1cc_ was between 17 and 18 Gy compared to a D_1cc_ < 17 Gy (Fig. 7D). When single-fraction radiation treatment for CCM was administered, the risk of PBE increased by more than 3-fold when D_1cc_ was >18 Gy compared to when D_1cc_ was between 17 and 18 Gy (Fig. 7D).

## 4 Discussion

Overall, the findings of the present study indicate that age independently predicts both nidus volume reduction and the occurrence of PBE after LINAC-based radiation therapy for non-brainstem CCMs. These findings suggest that caution should be exercised when performing LINAC-based radiotherapy for CCM in patients older than 55 years. This is because nidus volume reduction after radiotherapy for CCM may be less effective in older patients, while the possibility of PBE may be higher. In addition, high BED (≥119.67 Gy) was identified as an independent risk factor for PBE in non-brainstem CCM after radiotherapy. Additionally, a single fraction radiation dose of 16.5 Gy or greater was identified as an independent predictor for the reduction of nidus volume, while a dose of 17.5 Gy or greater posed an independent risk for PBE in patients with CCM undergoing LINAC-based radiation treatment. Consequently, we suggest that a single-fraction dose of approximately 17 Gy may be optimal for the treatment of non-brainstem CCM, particularly in patients younger than 55 years. To the best of our knowledge, no study has yet analyzed the association between D_1cc_, D_10cc_, and D_15cc_ in the surrounding normal brain tissue and the development of PBE after single-fraction radiotherapy in CCM. The results of our investigation showed that the D_1cc_ of the surrounding normal brain parenchyma had significant sensitivity and specificity for predicting PBE development after single-fraction radiotherapy for non-brainstem CCM. According to our findings, the risk of PBE increased when the maximum radiation dose delivered to a 1 cc volume of the surrounding normal brain tissue exceeded 17.3 Gy.

However, the efficacy of radiotherapy for CCM remains controversial. The role of radiotherapy in CCM is confounded by several difficult-to-control factors [2]. Therefore, to demonstrate that radiation treatment for CCM has beneficial effects, it is necessary to show that there is no increase in the annual risk of bleeding or symptomatic complications from the treatment itself. Several issues have arisen in the evaluation of the reduced incidence of hemorrhage in CCMs following radiation therapy. These include the need for extended observation periods to assess rebleeding rates, the uneven timing of hemorrhage development in untreated CCMs, biases in both selection and treatment, the potential for new CM formation, and the dynamic nature of CCMs [2,10,11,[15], [16], [17], [18]]. In this study, although neurologic deficits developed in some patients due to the insignificant intranidal hemorrhage taht often accompanies PBE, there were no cases of symptomatic cerebral hemorrhage during follow-up (median, 28.5 months) after radiotherapy for CCM. Recent research has shown that LINAC-based SRS is effective at preventing further bleeding in patients with non-brainstem CCMs [3]. Moreover, a recent meta-analysis indicated that SRS treatment of brainstem CCMs significantly lowers the risk of subsequent bleeding two years post-treatment [19]. Therefore, as it is difficult to prove that the bleeding rate is reduced after radiation treatment for CCM, we determined whether there was a nidus volume reduction in CCM on follow-up MR after radiotherapy to verify that radiation treatment for CCM does indeed exert a beneficial effect. CCM lesions that exhibited a reduction in size could be indicative of a lower rate of hemorrhage, with most lesions eventually resolving [11]. In addition, when the nidus volume increases after radiation therapy for CCM, new neurological deficits tend to occur, leading to a decreased quality of life [2]. Therefore, as described in the Methods section, we considered that radiation treatment had a beneficial effect on CCM in patients with a CCM nidus volume reduction of >20 % on the final follow-up MR compared with the MR at the time of radiation treatment [2].

According to our results, nidus volume reduction was more likely to occur in younger patients (<55 years) than in older patients after radiotherapy for non-brainstem CCM. However, the exact underlying pathophysiology remains unclear. However, it is now well accepted that successful obliteration of the nidus and a shorter treatment-obliteration interval of arteriovenous malformations (AVM) after radiation treatment are significantly associated with younger age [[20], [21], [22], [23], [24]]. Further, it has been postulated that the increased sensitivity of AVMs to radiation in younger patients might play a role in increasing the nidus obliteration rate [21,22]. Cells at different phases of the cell cycle exhibit different radiosensitivities. Cells in the late S phase are usually radioresistant, whereas those in the M phase (mitosis and cell division) are the most radiosensitive [25]. The cell division rates decrease with age [26]. Therefore, we hypothesized that the effect of radiation on vascular cells in the CCM nidus may be greater in younger patients, as there may be more vascular cells in M phase in younger patients than in older patients. In addition, the CCM is a lesion with dynamic size changes in which cell division occurs actively [11]. In addition, our multivariate analysis showed that PBE was more common in older patients treated with radiation for non-brainstem CCM. Furthermore, a previous study reported that the rebleeding rates from untreated CCMs were higher in younger patients [18]. Therefore, we believe that when performing radiotherapy for non-brainstem CCM, treatment of relatively young patients (55 years of age) is important to ensure a better treatment prognosis.

Ongoing discussions have focused on identifying the ideal radiation dosage for treating CCMs, balancing efficacy with the minimization of adverse effects. A previous investigation utilizing LINAC on non-brainstem CCMs demonstrated that a single radiation dose exceeding 16 Gy could successfully hemorrhagic episodes in affected individuals [3]. Similarly, our findings showed that when performing radiation therapy using LINAC for non-brainstem CCMs, a single-fraction radiation dose >16.5 Gy was effective for CCM nidus volume reduction. However, we also found that the probability of PBE was increased when the BED was >119.67 Gy, or the single fraction radiation dose was >17.5 Gy for the CCM treatment. In support of this finding, a previous study on CCM patients treated with a median margin radiation dose of 18 Gy reported that 59 % of patients developed a neurological deficit and 41 % of patients sustained a permanent neurological deficit due to PBE [27]. This complication rate is considerably high; therefore, we believe that the possibility of PBE increases when the single-fraction radiation dose is > 17.5 Gy when treating CCM.

As described above, in the present study, we found that the risk of PBE after single-fraction radiation therapy for CCM increased when the D_1cc_ of the surrounding normal brain tissue exceeded 17.3 Gy. Previous HyTEC (Hy Dose per Fraction, Hypofractionated Treatment Effects in the Clinic) reports recommend that the volume of normal brain receiving a radiation dose of 12 Gy or more during single fraction radiation therapy for AVMs should be ≤ 10 cc [28]. Consistent with HyTEC's recommendations, the maximum radiation dose administered to 10 cc of the surrounding normal brain was ≤12 Gy in all study patients, as displayed in Fig. 7A. For brain metastases <2 cm in size, HyTEC recommends delivering 18–24 Gy in a single fraction. However, brain metastases are commonly accompanied by PBE and degeneration of the surrounding brain tissue [29]. Therefore, the delivery of a relatively high dose to brain metastases is less likely to cause PBE development. Our results showed that among the D_1cc_, D_10cc_, and D_15cc_ of the normal brain after single-fraction radiotherapy for CCM, only the D_1cc_ significantly predicted the occurrence of PBE. Therefore, we hypothesized that calculating the D_1cc_ of the surrounding normal brain parenchyma is important for predicting PBE development after single-fraction radiotherapy in CCM. Owing to the compact size of CCMs, a significant radiation dose is precisely targeted to a small region of the brain. However, given the limited number of participants in this study, further research is required to confirm these findings.

Our study has several limitations. Firstly, the retrospective design meant that the follow-up duration and frequency of imaging varied significantly. In addition, the modest cohort size may have affected the statistical significance and reliability of the results. Consequently, additional studies with a larger number of participants are necessary to substantiate our conclusions. Finally, findings derived from a single institution may not be universally applicable or reproducible.

In conclusion, we found that patient age and radiation dose were the most important factors determining patient prognosis after LINAC-based radiotherapy for non-brainstem CCM. Our findings suggest that LINAC-based radiation therapy for non-brainstem CCMs with a single fraction radiation dose of >16.5 Gy but <17.5 Gy, or BED of approximately 120 Gy may be more effective at achieving nidus volume reduction while limiting side effects, particularly in patients under the age of 55 years. In addition, we believe that lowering D_1cc_ when administering single-fraction radiation therapy to patients with CCM would help to prevent the occurrence of PBE. However, due to the small sample size, our findings should be verified in future studies with larger cohorts.

## Declarations

**Ethical Approval and consent to participate:** The study received ethical approval and adhered to the regulations and guidelines by Hanyang University Guri Hospital Ethics Committee (approval no. 2023-03-034), South Korea and conformed to the tenets of the Declaration of Helsinki. The need for informed consent was waived due to the retrospective nature of the study by the Institutional Review Board of Hanyang University Guri Hospital Ethics Committee. All individual records were anonymized prior to the analysis. All methods were carried out in accordance with relevant guidelines and regulations.

## Data availability statement

The datasets used and/or analyzed during the current study are available from the corresponding author on reasonable request.

## Funding

This study was funded by the Basic Science Research Program through the 10.13039/501100003725National Research Foundation of Korea (NRF) funded by the Ministry of Science, ICT & Future Planning (NRF-2022R1F1A1063739).

## CRediT authorship contribution statement

**Sang Mook Kang:** Data curation. **Byeong Jin Ha:** Data curation. **Jin Hwan Cheong:** Supervision. **Je Il Ryu:** Supervision. **Yu Deok Won:** Supervision. **Myung-Hoon Han:** Writing – original draft, Visualization, Methodology, Investigation, Funding acquisition, Formal analysis, Conceptualization.

## Declaration of competing interest

The authors declare the following financial interests/personal relationships which may be considered as potential competing interests:Myung-Hoon Han reports financial support was provided by 10.13039/501100003725National Research Foundation of Korea (NRF). If there are other authors, they declare that they have no known competing financial interests or personal relationships that could have appeared to influence the work reported in this paper.
