# Supplementary material for: Identification of predictive factors for better outcomes in LINAC-based radiation treatment for cerebral cavernous malformation
Source: Heliyon. 2024 May 13;10(10):e31184. doi: 10.1016/j.heliyon.2024.e31184 (PMC11126848; doi:10.1016/j.heliyon.2024.e31184)
Supplement: Multimedia component 1 [file mmc1.docx]

**Supplementary Material**

**Identification of predictive factors for better outcome in LINAC-based radiation treatment for cerebral cavernous malformation**


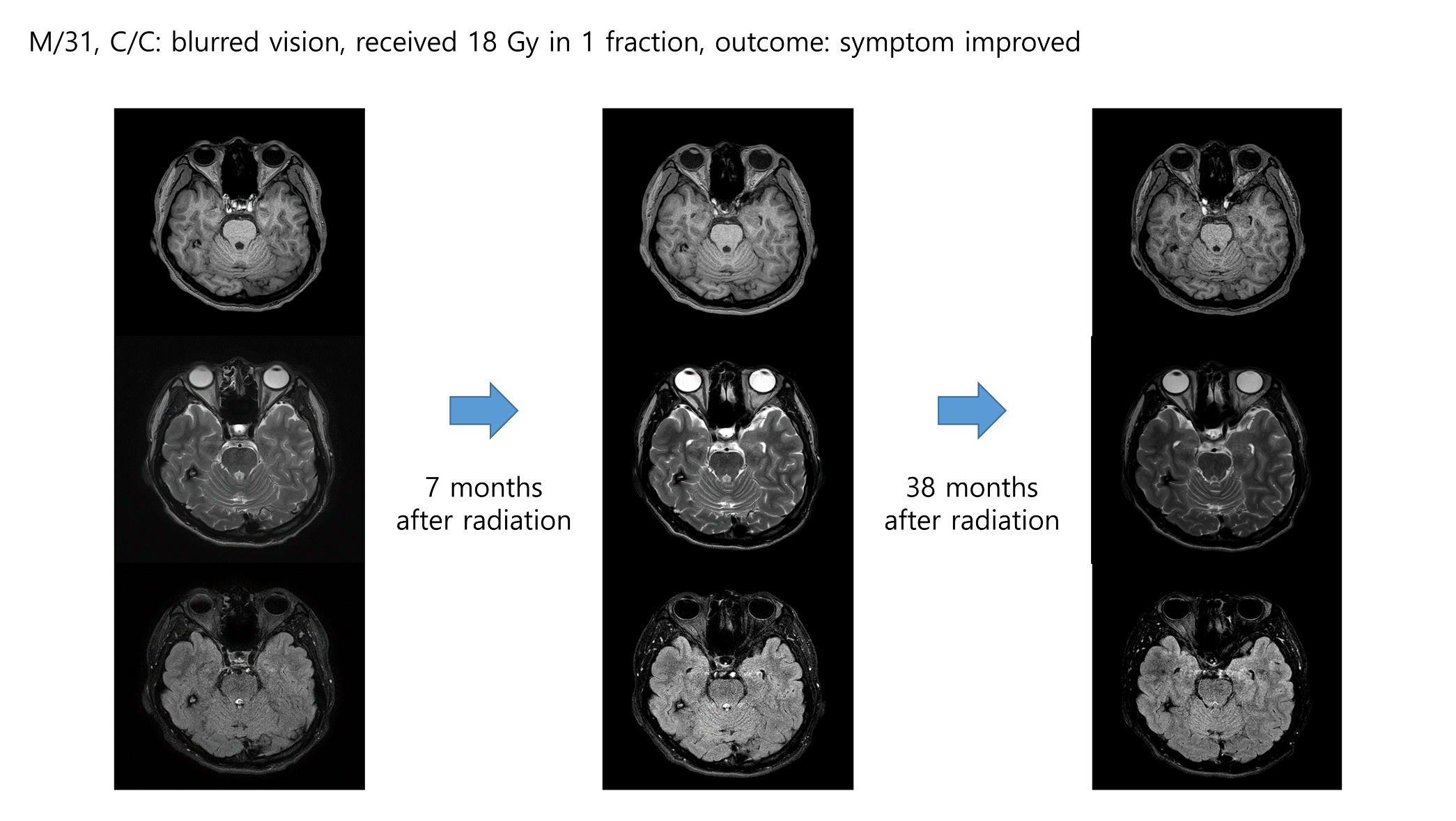


**Supplementary Fig. 1.** A 31-year-old male patient suddenly developed blurred vision. MRI revealed a CCM in the right temporal area and SRS treatment with 18 Gy was administered. Nidus volume reduction was observed 38 months after SRS treatment.

CCM, cerebral cavernous malformation; MRI, magnetic resonance imaging; SRS, stereotactic radiosurgery.


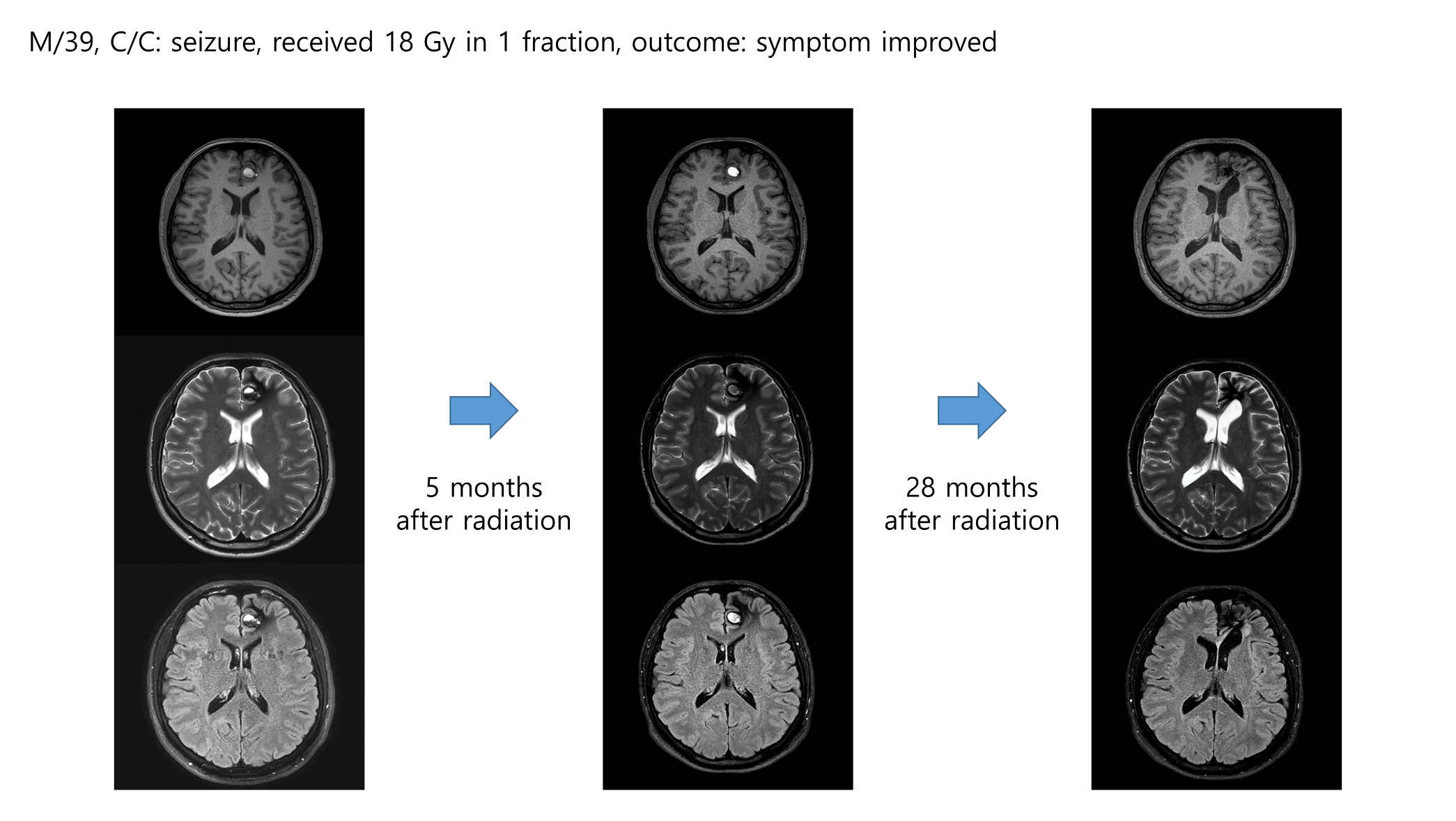


**Supplementary Fig. 2.** A 39-year-old male patient presenting with repeated seizure attacks. MRI revealed a cystic CCM in the left frontal lobe and SRS treatment with 18 Gy was administered. Nidus volume reduction was observed 28 months after SRS treatment.

CCM, cerebral cavernous malformation; MRI, magnetic resonance imaging; SRS, stereotactic radiosurgery.


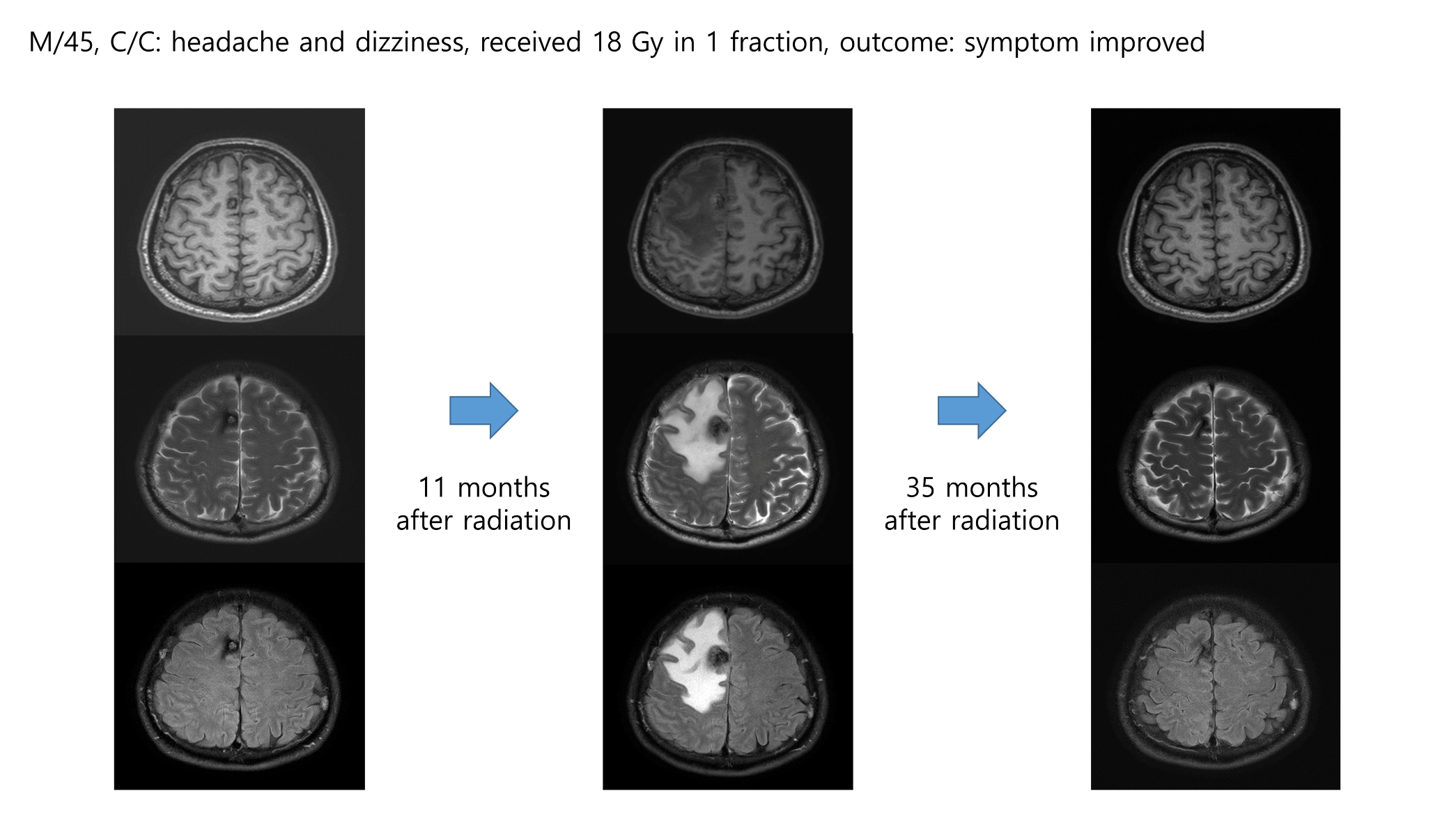


**Supplementary Fig. 3.** A 45-year-old male patient presenting with persistent headache and dizziness. MRI revealed a CCM in the right high frontal lobe and SRS treatment with 18 Gy was administered. Eleven months after radiation treatment, the patient presented with left hemiparesis and was admitted. MRI revealed an intranidal hemorrhage with severe PBE. The patient was treated with steroids for 4 months to control the brain edema. Thirty-five months after SRS treatment, the patient recovered completely and the last follow-up MRI showed CCM nidus volume decrease and no PBE.

CCM, cerebral cavernous malformation; MRI, magnetic resonance imaging; PBE, perilesional brain edema; SRS, stereotactic radiosurgery.


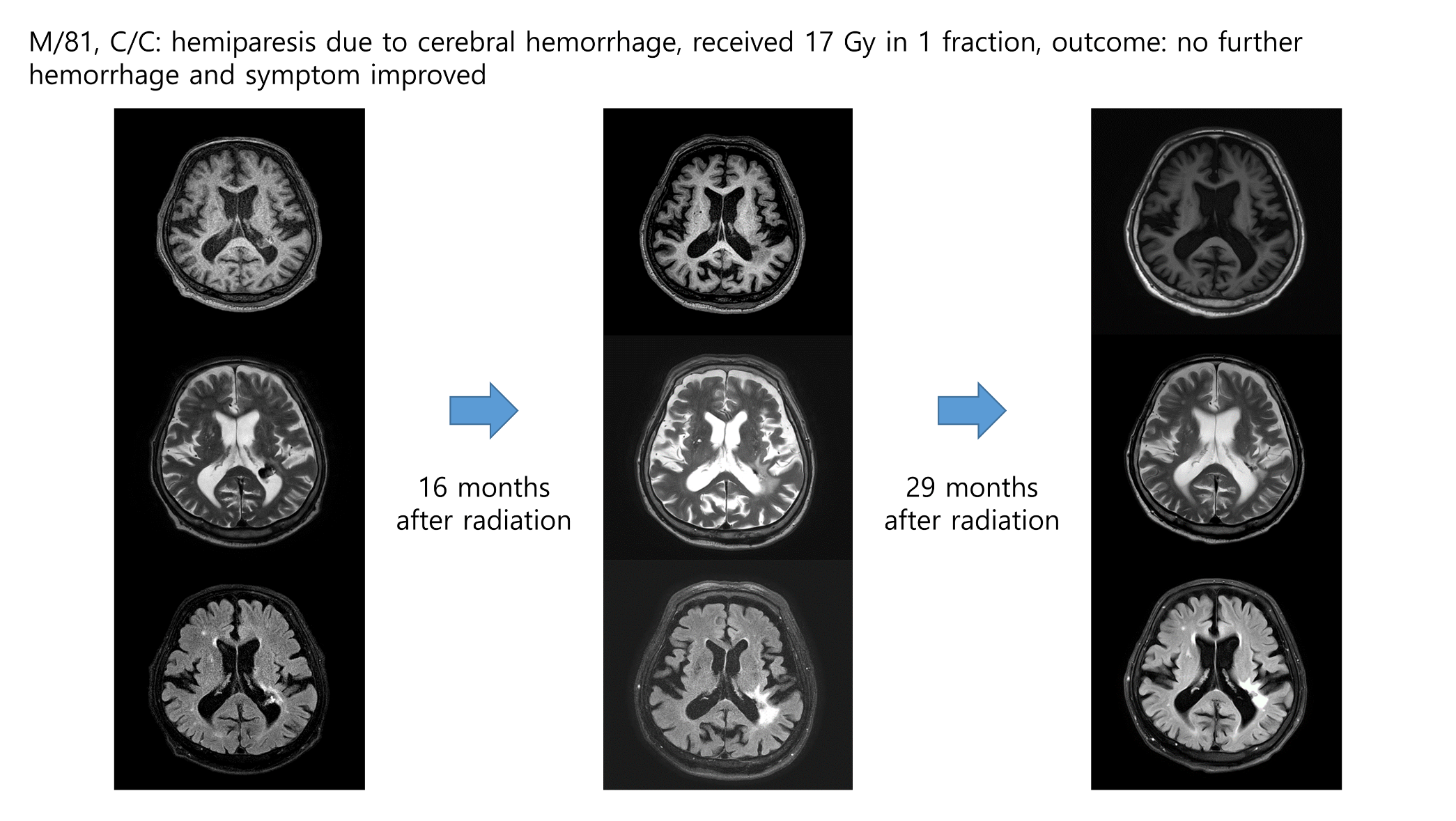


**Supplementary Fig. 4.** An 81-year-old male patient presenting hemiparesis due to cerebral hemorrhage. MRI revealed a CCM located in the left posterior paraventricular area and SRS treatment with 17 Gy was administered. Sixteen months after SRS treatment, follow-up MRI showed nidus volume decrease with asymptomatic PBE. The patient was treated with steroid for 3 months. Twenty-nine months after radiation treatment, the patient’s symptoms improved and the last follow-up MRI showed CCM nidus volume decrease and persistent but slightly decreased asymptomatic PBE.

CCM, cerebral cavernous malformation; MRI, magnetic resonance imaging; PBE, perilesional brain edema; SRS, stereotactic radiosurgery.


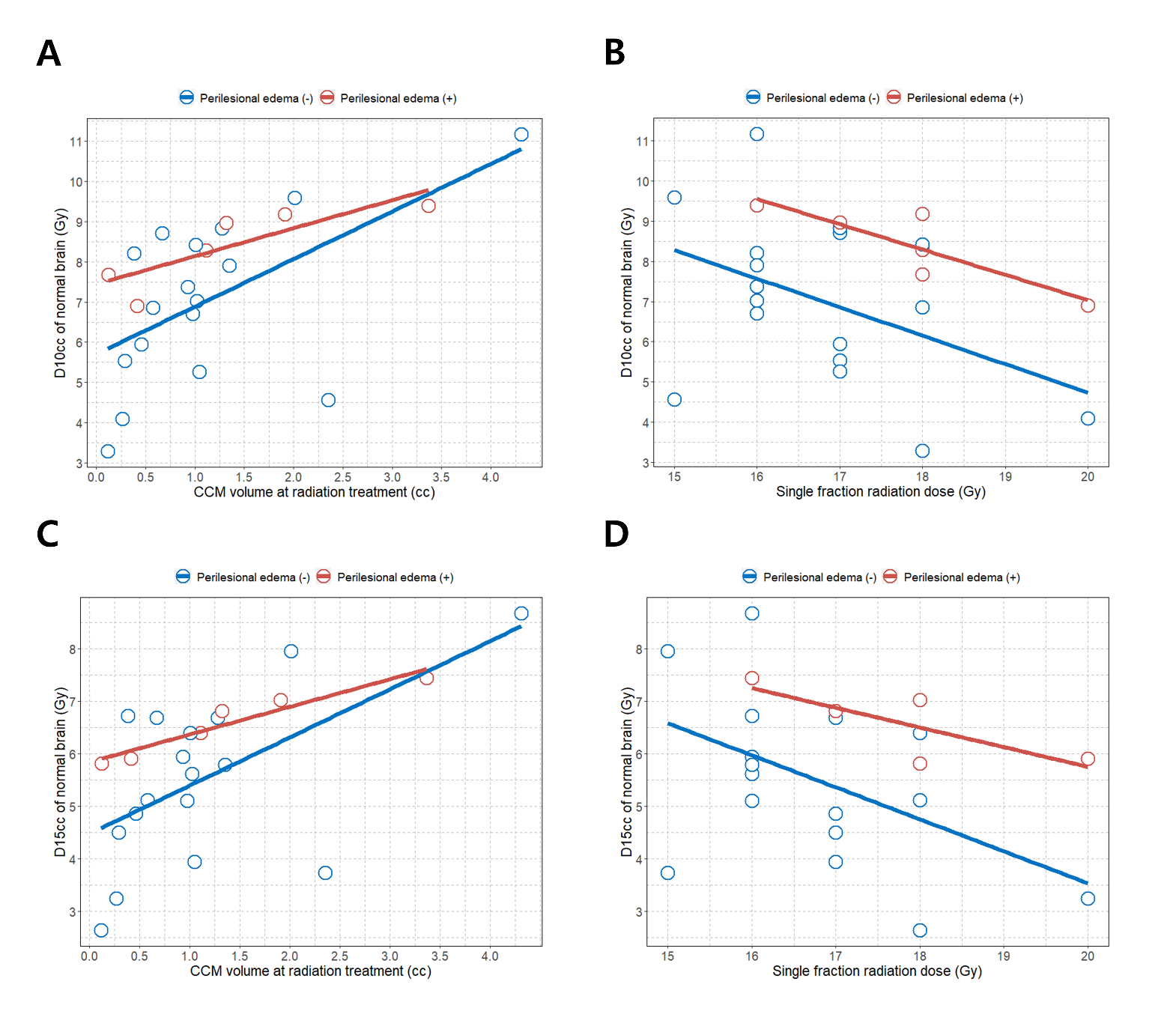


**Supplementary Fig. 5.** (A) Scatter plot with linear regression lines showing the association between CCM volume during radiation treatment and D_10cc_ of normal brain parenchyma according to PBE presence/absence; (B) Scatter plot with linear regression lines showing the association between single fraction radiation dose and D_10cc_ of normal brain parenchyma according to PBE presence/absence; (C) Scatter plot with linear regression lines showing the association between CCM volume at radiation treatment and D_15cc_ of normal brain parenchyma according to PBE presence/absence; (D) Scatter plot with linear regression lines showing the association between single fraction radiation dose and D_15cc_ of normal brain parenchyma according to PBE presence/absence;

CCM, cerebral cavernous malformation; D_1cc_, maximum dose delivered to a volume of 1cc of surrounding normal brain parenchyma; D_10cc_, maximum dose delivered to a volume of 10cc of surrounding normal brain parenchyma; D_15cc_, maximum dose delivered to a volume of 15cc of surrounding normal brain parenchyma; PBE, perilesional brain edema.

**Supplementary Table 1.** Univariate Cox regression analyses of nidus volume reduction and perilesional brain edema after LINAC-based radiation treatment for CCM based on predictive factors in all patients and patients received SRS

| **All CCMs (n=31)** | | | | |
| --- | --- | --- | --- | --- |
|  | Nidus volume reduction | | Perilesional brain edema | |
| Variable | HR (95% CI) | p | HR (95% CI) | p |
| Sex |  |  |  |  |
| Male | Reference |  | Reference |  |
| Female | 0.79 (0.30–2.06) | 0.627 | 1.69 (0.42–6.80) | 0.457 |
| Age  (per 1–year increase) | **0.96 (0.93–0.99)** | **0.019** | 1.02 (0.96–1.08) | 0.573 |
| Location |  |  |  |  |
| Supratentorial | Reference |  | Reference |  |
| Infratentorial | 0.65 (0.15–2.88) | 0.572 | 1.19 (0.15–9.75) | 0.872 |
| BED |  |  |  |  |
| < 108.13 Gy | Reference |  | N/A |  |
| ≥ 108.13 Gy | 1.62 (0.64–4.07) | 0.308 |  |  |
| < 119.67 Gy | N/A |  | Reference |  |
| ≥ 119.67 Gy |  |  | 4.21 (1.00–17.75) | 0.050 |
| Zabramski classification | 1.43 (0.94–2.16) | 0.094 | 1.30 (0.67–2.54) | 0.438 |
| CCM volume  (per 1 cc increase) | 1.08 (0.97–1.20) | 0.153 | 1.10 (0.95–1.26) | 0.217 |
| Fractionation |  |  |  |  |
| SRS | Reference |  | Reference |  |
| hf-SRS | **3.51 (1.22–10.09)** | **0.020** | 2.05 (0.41–10.36) | 0.385 |
|  | | | | |
| **CCMs received single fraction radiotherapy (n=23)** | | | | |
|  | Nidus volume reduction | | Perilesional brain edema | |
| Variable | HR (95% CI) | p | HR (95% CI) | p |
| Sex |  |  |  |  |
| Male | Reference |  | Reference |  |
| Female | 0.54 (0.15–1.94) | 0.345 | 1.66 (0.34–8.26) | 0.534 |
| Age  (per 1–year increase) | 0.96 (0.92–0.99) | 0.024 | 1.03 (0.96–1.10) | 0.408 |
| Location |  |  |  |  |
| Supratentorial | Reference |  | Reference |  |
| Infratentorial | 0.80 (0.18–3.68) | 0.778 | 1.52 (0.18–13.01) | 0.705 |
| Radiation dose |  |  |  |  |
| < 17 Gy | Reference |  | N/A |  |
| ≥ 17 Gy | **4.58 (1.02–20.50)** | **0.047** |  |  |
| < 18 Gy | N/A |  | Reference |  |
| ≥ 18 Gy |  |  | 5.08 (0.92–28.00) | 0.062 |
| CCM volume  (per 1 cc increase) | 0.97 (0.53–1.78) | 0.919 | 1.13 (0.57–2.24) | 0.717 |

LINAC, linear accelerator; CCM, cerebral cavernous malformation; SRS, stereotactic radiosurgery; HR, hazard ratio; CI, confidence interval; BED, biologically equivalent dose; N/A, not available.
